# Survey of Crimean-Congo Hemorrhagic Fever Enzootic Focus, Spain, 2011–2015

**DOI:** 10.3201/eid2506.180877

**Published:** 2019-06

**Authors:** Ana Negredo, Miguel Ángel Habela, Eva Ramírez de Arellano, Francisco Diez, Fátima Lasala, Pablo López, Ana Sarriá, Nuria Labiod, Rafael Calero-Bernal, Miguel Arenas, Antonio Tenorio, Agustín Estrada-Peña, Maria Paz Sánchez-Seco

**Affiliations:** National Center of Microbiology, Madrid, Spain (A. Negredo, E. Ramírez de Arellano, F. Diez, F. Lasala, P. López, N. Labiod, A. Tenorio, M.P. Sánchez-Seco);; University of Extremadura, Cáceres, Spain (M.Á. Habela, R. Calero-Bernal);; La Paz Hospital, Madrid (A. Sarriá); University of Vigo, Pontevedra, Spain (M. Arenas);; University of Zaragoza, Zaragoza, Spain (A. Estrada-Peña)

**Keywords:** Crimean Congo hemorrhagic fever virus, CCHFV, viruses, Crimean-Congo hemorrhagic fever, Hyalomma spp. ticks, reverse transcription PCR, molecular epidemiology survey, phylogenetic analysis, vector-borne infections, tick-borne infections, endemic circulation, zoonoses, enzootic focus, Spain

## Abstract

During 2011–2015, we conducted a Crimean-Congo hemorrhagic fever virus (CCHFV) survey in captured ticks that were feeding mainly on wild and domestic ungulates in Spain, where presence of this virus had been reported previously. We detected CCHFV RNA in *Hyalomma lusitanicum* and *H. marginatum* ticks for 3 of the 5 years. The rate of infected ticks was 2.78% (44/1,579), which was similar to those for other countries in Europe with endemic foci for CCHFV (Kosovo, Bulgaria, and Albania). These data confirm the established spread of CCHFV into western Europe. Phylogenetic study of the small RNA segment showed Africa-3 clade as the only genotype identified, although we observed cocirculation of genetic variants during 2011 and 2015. We could not rule out genetic reassortments because of lack of sequence data for the medium and large RNA segments of the virus genome.

Crimean-Congo hemorrhagic fever virus (CCHFV), an RNA virus of the family *Nairoviridae*, is the most widespread tickborne virus affecting humans. It causes Crimean-Congo hemorrhagic fever (CCHF), which has a high mortality rate in humans (*1*) in many countries in Asia, the Middle East, eastern Asia, Africa, and Europe (*2**,**3*). Recently, CCHF has been detected in Spain, indicating its spread into western Europe (*4**,**5*).

CCHF is a zoonosis maintained between ticks and vertebrate animals. Ticks are both the vector and the natural reservoir of CCHFV. Ticks transmit CCHFV to large and small mammals, which act as amplifier hosts without signs of illness. Birds are predominantly refractory to CCHFV infection (*6**–**8*). The virus has been isolated from several genera and species of ixodid ticks (*9*). However, most data are for ticks collected while feeding, a method that provides an unrealistic view of the actual species vectors (*10*).

The main vectors involved in CCHFV transmission are ticks of the genus *Hyalomma* (*11**–**14*). Immature ticks acquire the virus by feeding on infected small vertebrates or by the transovarial route. Once infected, they remain infected throughout their development and, when they are mature, transmit the infection to large animals, such as livestock. Transovarian and venereal transmission pathways have also been demonstrated (*2*). Another transmission mechanism is cofeeding, which results from spreading of the virus in tick saliva directly to other ticks feeding nearby (*13*).

In disease-endemic areas, CCHF is sporadic and is transmitted to humans by tick bites or contact with viremic animals or humans and occurs mainly in remote or agricultural regions (*15**–**21*). CCHF is seasonal in association with the life cycle and activity levels of local tick populations. CCHF outbreaks and nosocomial transmission has also been reported most often when an infectious case is not suspected early enough to permit use of proper containment protocols (*22*). Persons at risk are most frequently found among farmers and their families, as well as slaughterhouse and healthcare workers, veterinarians, and military personnel (*23*).

Only 2 autochthonous human cases of CCHF have been reported in Spain in an episode with tick and nosocomial transmission. The cases occurred in 2016, and the viruses isolated from these patients were included in the Africa-3 clade (genotype III) (*5**,**24*), the same clade that was found in local ticks (*4**,**25*) and 1 of the 6/7 genetic clades in which CCHFV strains are grouped on the basis of small (S) RNA segment sequence homology (*26**,**27*). Previously, the CCHFV genome was detected in ticks collected in 2010 from red deer in Cáceres, Spain (*4*), which is 300 km from the area (Ávila Province) where the index case-patient acquired the disease through tick bite transmission. After autochthonous human cases were detected, a surveillance study on ticks was conducted to investigate the spread of CCHFV in Spain. Results showed 4 geographic regions with CCHFV-positive results (Figure 1) and the presence of CCHFV in 128 (3.2%) of 3,959 pools of ticks collected from wild and domestic animals (*28*). All CCHFV-positive ticks were collected while they were feeding on wild animals.

**Figure 1:**
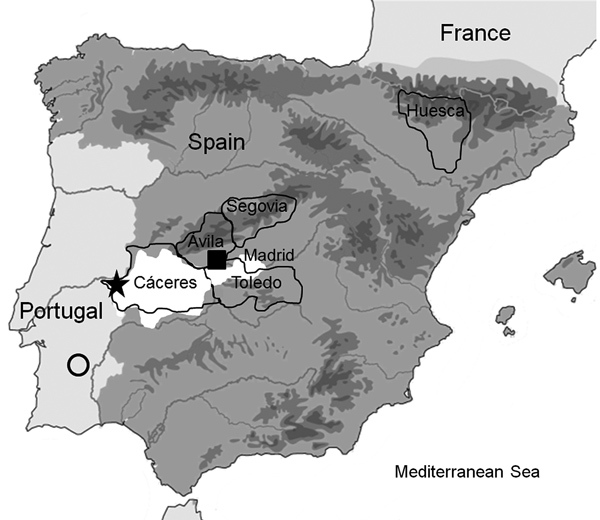
Study site locations in the Iberian Peninsula in which Crimean-Congo hemorrhagic fever virus was detected: Cáceres, Toledo, Segovia, and Huesca Provinces. Square shows presence of CCHFV in humans bitten by a tick, star shows presence of CCHFV in ticks with positive results by PCR, circle indicates region where serum samples positive for CCHFV were detected in Portugal, and white area shows regions in 4 localities (Cáceres, Ávila, and Toledo Provinces and Madrid) in Spain where CCHFV-positive ticks had been previously identified.

These data highlighted the emerging threat for CCHF in Spain and potentially all of western Europe. A new case of CCHF also linked to tick bite transmission occurred in August 2018 in Spain. A study of the presence of CCHFV in Spain was initiated in 2011 to analyze principally its circulation in the region of original detection. We report the results of a survey covering 5 years of collection.

## Materials and Methods

### Study Area and Tick Collections

Tick collection in Cáceres Province (southwestern Spain, 37°N–41°N, 3°W–7°W) (Figure 1) was focused on adult ticks infesting red deer (*Cervus elaphus*) and, to a lesser extent, other mammals, such as fallow deer (*Dama dama*), red foxes (*Vulpes vulpes*), and wild boars (*Sus scrofa ferus*), legally hunted in privately owned estates during October–February. Sampling areas are located within typical Mediterranean *Quercus* forest in several protected areas and reserves (Tajo International Natural Park and Sierra de San Pedro Mountain Range), which constitute an optimum ecosystem for wild ungulates. Collection of ticks from cattle was also conducted in Cáceres Province. We directly shipped ticks collected at room temperature to the Parasitology Laboratory of the Faculty of Veterinary Medicine at the University of Extremadura (Cáceres). Ticks were identified to species within 1–2 days upon arrival, and frozen at −65°C while alive until isolation of RNA. Tick collection in Segovia Province (41.21°N, 3.59°W) was conducted during the sheep shearing process, and adult ticks captured were identified in the Spanish National Center of Microbiology. Tick collections on the ground were also performed in Huesca (42.21°N, 0.13°W) and Toledo (39.23°N, 4.29°W) Provinces. We captured mainly adult ticks and identified them in the Animal Pathology Department of the Faculty of Veterinary Medicine at the University of Zaragoza. All ticks were sent in dry ice to the Spanish National Center of Microbiology (Madrid, Spain) for processing.

### Extraction of RNA from Tick Species

Ticks were washed twice with water and once with ethanol and then individually crushed by using a plastic homogenizer in 500 µL AVL buffer (QIAGEN, https://www.qiagen.com). We conducted homogenization in a Biosafety Level-3 laboratory. After homogenization, we stored 250 µL of the sample in AVL buffer at −80°C and used 250 µL for total extraction of RNA with the QIAamp Viral RNA Mini Kit (QIAGEN), according to the recommendations of the manufacturer. RNA was eluted in 60 µL of RNAase-free water and stored at −80°C until needed.

### Reverse Transcription PCR

We used an in-house nested reverse transcription PCR specific for the S genome segment (*5*). We individually analyzed ticks.

### DNA Sequencing

We purified amplified DNA by using an Illustra ExoProStar Kit (GE Healthcare Life Sciences, https://www.gelifesciences.com). We sequenced double-stranded DNA directly by using the Sanger chain-termination method and the BigDye Terminator v3.1 Cycle Sequencing Kit Protocol and the ABI PRISM 3700 DNA Analyzer (Applied Biosystems, https://www.thermofisher.com). We used sequencing primers CrCon2+ and CrCon2–, the same used in the nested PCR (*5*). We assembled consensus sequences of each segment and analyzed them by using the SeqMan Program in the Lasergene Package (https://www.dnastar.com).

### Phylogenetic Analysis

We aligned nucleotide sequences by using Muscle in MEGA version 7 (https://www.megasoftware.net). Because recombination can bias phylogenetic tree reconstruction, we analyzed recombination in the alignment with the Recombination Detection Program (http://web.cbio.uct.ac.za/~darren/rdp.html) and the Hypothesis Testing Using Phylogenies Program (http://hyphy.org). We did not detect genetic signatures of recombination.

We generated a maximum clade credibility (MCC) genealogy by using BEAUTi and BEAST (*29*). We ran the Markov chain Monte Carlo method for 100 million generations, sampling every 1,000 steps, and analyzed convergence with Tracer (http://www.beast2.org) to ensure that effective sample sizes were >200. We applied the uncorrelated lognormal clock model, the general time-reversible matrix with gamma distribution and proportion of invariable sites substitution model, and the exponential tree prior. We summarized reconstructed trees by applying the MCC approach implemented in TreeAnnotator version 1.8.4 (http://beast.community/treeannotator), used the mean value as the node height, and discarded the first 10% of the generated trees as burn-in. We visualized the MCC tree by using FigTree version 1.4.3 (http://tree.bio.ed.ac.uk). We deposited nucleotide sequences with lengths >200 nt obtained with primers CrCon1+ and CrCon 1– (*5*) in the European Molecular Biology Laboratory/GenBank databases (accession nos. MH337845 and MH337846).

## Results

We collected 1,579 ticks in 4 geographic regions in Spain (Figure 1), 206 from vegetation and 1,373 from animals. Of ticks collected from animals, 1,329 (96.79%) were from wild animals and 44 (3.35%) were from domestic animals.

Collected ticks that we could identify belonged to 1 of 4 genera: *Rhipicephalus* (46, 2.91%), *Hyalomma* (1,317, 83.40%), *Dermacentor* (1, 0.06%), or *Ixodes* (3, 0.18%); 212 ticks could not be identified. Most ticks were *H. lusitanicum* (1,079, 68.33%) and *H. marginatum* (238, 15.07%) (Table). *H. lusitanicum* ticks were obtained mainly from wild animals (1,063/1,329), and *H. marginatum* ticks (206/238) were collected principally from vegetation.

**Table:** Characteristics of ticks positive for Crimean-Congo hemorrhagic fever virus, Spain, 2011–2015*

Year (no. ticks)	Province (no. ticks)	Sampling site	Collection site or host	Tick species	No. positive ticks/no. tested (%)
2011 (474)	Toledo (78)	31	Ground	*Hyalomma marginatum*	0/78
	Huesca (128)	32	Ground	*H. marginatum*	0/128
	Cáceres (268)				
		1	Deer	*H. lusitanicum*	**8/139 (5.75)**
		2	Deer	*H. lusitanicum*	**3/35 (8.57)**
		3	Deer	*H. marginatum*	0/4
		4	Deer	Unknown	0/20
		5	Red fox	Unknown	0/3
		6	Cattle	Unknown	0/2
		7	Deer	*H. lusitanicum*	0/24
		8	Deer	Unknown	0/3
		9	Deer	Unknown	0/7
		10	Deer	Unknown	0/20
		11	Deer	*Rhipicephalus bursa* nymphs	0/11
2012 (48)	Cáceres (27)	1	Deer	*H. lusitanicum*	0/27
	Segovia (21)	33	Cattle/sheep	*Rhipicephalus* sp.	0/21
2013 (201)	Cáceres (201)				
		1	Deer	*H. lusitanicum*	**13/68 (19.11)**
		2	Deer	Unknown	0/5
		10	Deer	*H. lusitanicum*	0/53
		12	Deer	*H. lusitanicum*	0/24
		13	Deer	*R. bursa*	0/5
		14	Deer	*H. marginatum*	0/23
		15	Deer	Unknown	0/6
		16	Deer	Unknown	0/17
2014 (272)	Cáceres (272)				
		1	Deer	*H. lusitanicum*	0/57
		4	Deer	Unknown	0/41
		15	Deer	Unknown	0/17
		17	Deer and wild boar	Unknown	0/18
		18	Deer and wild boar	*H. lusitanicum*	0/20
		19	Deer	Unknown	0/31
		20	Deer	*H. lusitanicum*	0/35
		21	Deer and wild boar	Unknown	0/19
		22	Deer	*H. lusitanicum*	0/18
		23	Deer	*H. lusitanicum*	0/16
2015 (584)	Cáceres (584)				
		1	Deer	*H. lusitanicum*	**3/66 (4.54)**
		2	Deer	*H. lusitanicum*	**11/120 (9.16)**
		4	Deer	*H. lusitanicum*	0/25
		18	Deer	*H. lusitanicum*	0/40
		19	Deer	30 *H. lusitanicum*, 1 *R. bursa*	0/31
		23	Deer	34 *H. lusitanicum*, 1 *Ixodes ricinus*	**1/35 (2.85)**
			Cattle	16 *H. lusitanicum*, 5 *H. marginatum*	**3/21 (14.28)**
		24	Deer	14 *H. lusitanicum*, 8 *R.bursa*, 1 *Dermacentor* sp.	0/23
		25	Fallow deer	6 *H. lusitanicum*, 2 *I. ricinus*. 3 unknown	0/11
		26	Deer	*H lusitanicum*	0/32
		27	Deer	*H. lusitanicum*	1/36 (2.77)
		28	Deer	*H. lusitanicum*	1/45 (2.22)
		29	Deer	*H. lusitanicum*	0/72
		30	Deer	*H. lusitanicum*	0/27
Total					**44/1579 (2.78)**

*Ticks positive for Crimean-Congo hemorrhagic fever virus are indicated in bold. Animals tested were red fox (*Vulpes vulpes*), red deer (*Cervus elaphus*), fallow deer (*Dama dama*), and wild boar (*Sus scrofa ferus*).

We detected CCHFV RNA in 44 (2.78%) of 1,579 ticks collected (Table). All CCHFV-positive ticks were detected in Cáceres (3.25%; 44/1,352). We collected ticks in 31 localities within Cáceres; 5 (16.12%) localities (1, 2, 23, 27, and 28) had virus-positive ticks. Rates for CCHV-positive ticks were 8.79% in locality 1, 9.03% in locality 2, 7.14% in locality 23, 2.77% in locality 27, and 2.22% in locality 28. CCHFV positive ticks were collected during 2011, 2013, and 2015. In Cáceres, the rate of CCHFV in ticks was 4.1% in 2011, 6.4% in 2013, and 3.42% in 2015.

A total of 43 *H. lusitanicum* ticks and 1 *H. marginatum* tick were positive for CCHFV (Table); 41 of 43 CCHFV-positive *H. lusitanicum* ticks were collected while feeding on red deer, and CCHFV-positive *H. marginatum* ticks were collected from 1/6 bovine animals analyzed in locality 23 (Table). Six *H. lusitanicum* ticks and 1 *H. marginatum* tick were found on this animal. Of these ticks, 1 *H. lusitanicum* male and 1 *H. marginatum* female were positive for CCHFV. This animal might not have been viremic, which would explain why only 2 of 6 ticks were infected by the co-feeding transmission mechanism, in which virus present in tick saliva can spread directly to other ticks feeding nearby. In locality number 23, CCHFV-positive *H. lusitanicum* ticks were detected on a second cow and 1 red deer.

We obtained sequences from 43 of 44 CCHFV-positive ticks. CCHFV sequences from Spain had the highest identities (90.6%–99.6%) with the Sudan AB-1–2009 strain in the analyzed fragment of the S RNA segment.

**Figure 2:**
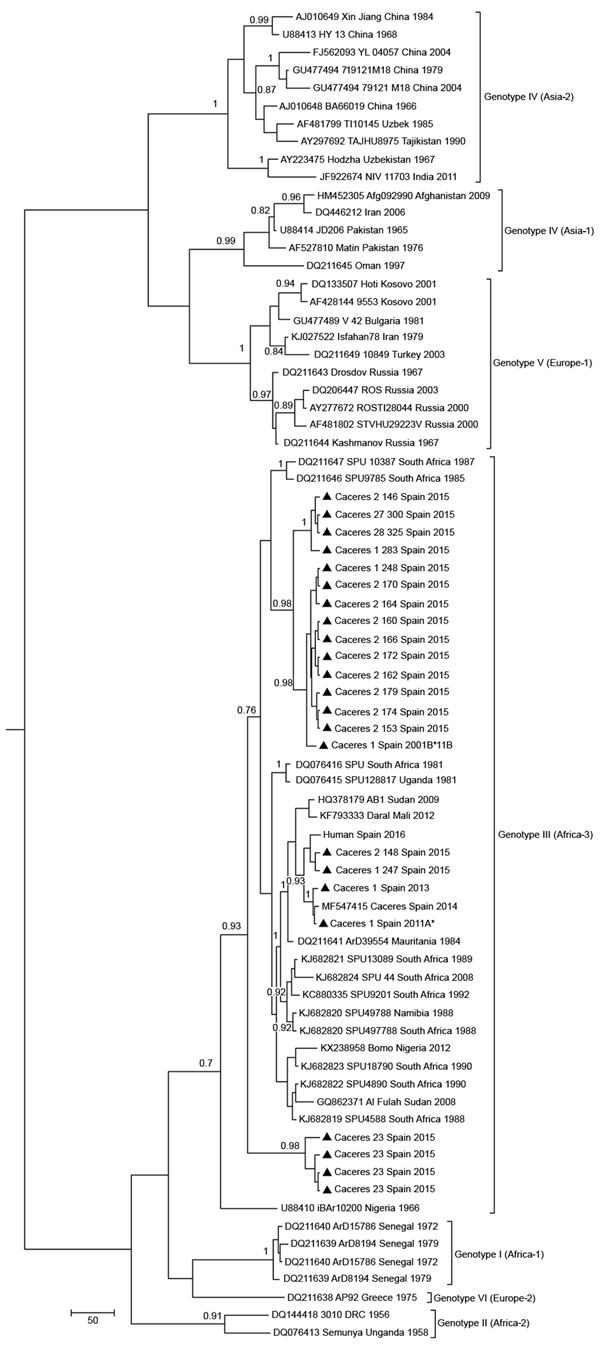
Maximum clade credibility genealogy based on partial (175-nt) sequences of the Crimean-Congo hemorrhagic fever virus small RNA segment of viruses from Spain and reference sequences. Numbers along branches indicate posterior probability values for the groups (values <0.70 are not shown). Triangles indicate newly sequenced strains from Spain reported in this study, which are identified by geographic origin, sampling site, and sampling year. Other sequences are indicated by GenBank accession number, strain, geographic origin, and sampling year. Sequences from this study indicated with an asterisk are included in European Molecular Biology Laboratory/GenBank databases. Genotypes are indicated in roman numerals and named according to Carroll et al. (*30*). Equivalent clade nomenclature is listed according to Chamberlain et al. (*31*) and indicated at right. Scale bar indicates nucleotide substitutions per site.

Phylogenetic analyses based on a 175-bp fragment of the S RNA segment separated CCHFV into 7 well-supported groups in association with the geographic distribution Asia 1, Asia 2, Africa 1–3, and Europe 1 and 2. All sequences grouped with clade Africa-3 (genotype III) viruses from South Africa and West Africa (Figure 2) and showed 4 groups with identities of 100%. One group (A) was formed by 4 sequences, the second group (B) by 12, the third group (C) by 24, and the fourth group (D) by 4. For each group, the percentage of diversity was 0.07%–0.09%. Bayesian analysis showed that estimated the time to the most recent common ancestor (tMRCA) of lineage A was ≈1979 (95% highest posterior density [HPD] 1968–1984), which diverged before lineages B (1999; 95% HPD, 1986–2009), C (2004; 95% HPD, 1992–2014), and D (1996; HPD 1973–2013). Use of the 175-nt fragment to calculate the tMRCA of lineage A was supported by results reported by Cajimat et al. (*25*), who used the complete S segment (1965; 95% HPD 1948–1980).

## Discussion

An ongoing surveillance study of CCHFV in ticks was conducted because this virus was detected in Spain in 2010 (*4*). Thus, ticks were collected during 2011–2015, mainly in wild animals in the region where CCHFV was detected (Cáceres) and also in other regions (Huesca and Toledo) that are colonized by *Hyalomma* spp., or that have an abundance of domestic ungulates (Segovia). We detected CCHFV in Cáceres in 3 of the 5 years, indicating the resilience of a CCHFV focus in Spain and confirming the established spread of CCHFV into western Europe.

The rate of CCHFV-infected ticks determined in this study (2.78%, 44/1,579) was similar to those for other countries in Europe that have enzoonotic foci, such as Kosovo (3.6%, 40/1,102) (*12*), Bulgaria (2%–4.83%) (*32*), and Albania (3%) (*23*). Despite these data, no human cases were documented in Spain during the years of the study. This situation shows that human cases probably occur rarely, even though the virus is quite active in host-seeking ticks in the area. We detected CCHFV in *Hyalomma* ticks collected on cattle and deer in Cáceres in 5 of 31 hunting areas, 2 of which have repeatedly been found to be positive for CCHFV. In the maintenance of active CCHFV foci, habitat fragmentation, such as in the Cáceres region studied, might lead to isolated populations of ticks and hosts producing an amplification cycle in which ticks feed on infected hosts (*33*). However, birds carrying infected ticks on their surface or infected small mammals, such as brown hares and rabbits, can spread the virus to new areas. In August 2016, in a rural area of Ávila, Spain (Figure 1), where no evidence of CCHFV circulation was previously described and that is 300 km from the CCHFV enzoonotic focus in Cáceres, a human infection occurred after a tick bite (*5*).

Our study spanned 5 years; virus was detected during 2011, 2013, and 2015. In 2012, the number of samples collected was low. During 2014, our laboratory obtained 2 CCHFV-positive ticks that had low viral loads that could not be confirmed, although virus circulation during that year in Cáceres was demonstrated by Cajimat et al. (*25*). This finding reflects intermittent activity of the CCHFV foci in Cáceres, which becomes periodically apparent as described in other countries (*13**,**34*). In southern Portugal, which is close to this region (Figure 1), antibodies against this virus were detected in 2 human serum samples during 1980 (*35*), suggesting silent circulation of the virus in the Iberian Peninsula. Introduction of the virus into Spain has been calculated to have occurred ≈50 years ago (*25*), which was corroborated by our study.

In our study, of the 4 genera of ticks collected, only *Hyalomma* ticks were found to harbor CCHFV (Table). All CCHFV-positive ticks were collected while feeding on ruminants, red deer, and cattle. All except 2 CCHFV-positive ticks, 1 *H. lusitanicum* and 1 *H. marginatum,* were collected on red deer. This finding confirmed that this tick–host relationship had a primary role in maintenance of CCHFV enzootic cycles in Spain and the role of red deer as a prominent host. The CCHFV-positive *H. marginatum* tick was collected from a cow along with an *H. lusitanicum* tick. Presumably, these ticks were infected by a cofeeding transmission mechanism in nonviremic animals because only 2 of 6 ticks feeding on the same animal were positive for CCHFV. Until now, CCHFV-positive *H. marginatum* ticks have not been detected in Spain, although this species is considered to be the main reservoir of CCHFV in Europe. This tick species can increase the risk for human cases of infection in Spain because of its proximity to human populations when compared with *H. lusitanucum* ticks. In addition, CCHFV-positive *H. marginatum* ticks have been captured in domestic animals that have close contact with humans. Previous studies in Spain that analyzed 2,500 ticks (*28*) and 1,408 ticks (*36*) collected from domestic ungulates did not report any CCHFV-infected ticks. Our results clearly show that the virus is active in the Cáceres region and that cattle might play a major role in transmission of the virus to new generations of ticks that could act as bridge vectors.

CCHFV sequences from Spain isolated during this study grouped with clade Africa-3. This clade contains strains separated by large spatial distances, mainly from Mauritania to Senegal, Nigeria, Sudan, and South Africa, suggesting that virus is most likely spread by migratory birds that transmit infected ticks or by secondary introductions after importation of commercial livestock. The presence of the Africa-3 clade in Europe must be considered because of the increased risk for its spread to other countries in Europe where *Hyalomma* ticks are present. In Greece, the Africa-3 clade was detected in ticks from migratory birds in the Greek archipelago (*37*), although this clade has not been detected in continental Greece. Virus sequences found in ticks in Spain are more closely related to CCHFV strains from central-northern Africa, a finding that supports the theory that the virus was spread northward by migrating birds. Moreover, the Africa-3 clade was detected recently in ticks from migratory birds in Morocco (*38*).

Although it is recognized that 175 nt is a small genetic fragment for robust comparisons between closely related viruses, phylogenetic analyses of CCHFVs showed 4 well-supported clusters (with bootstrap values >0.7 and p distances among clusters of 0.07%–0.09%). We detected cocirculation of different clusters: 2 clusters during 2011 and 4 during 2015. Phylogenetic analysis showed that the tMRCA of the 4 CCHFV variants diverged to produce 2 lineages, 1 containing variants A, B, and C and the other containing variant D. Whether this divergence occurred in Africa or in Spain cannot be determined with the limited sequence data currently available. The estimated tMRCA of lineage A was ≈1979 and it diverged before lineages B (1999), C (2004), and D (1996). Use of the 175-nt fragment to estimate the tMRCA of lineage A is supported by results from another study (*25*), in which the complete S RNA segment was used (1965; 95% HPD 1948–1980). Circulation of genetic variants of the same genotype could indicate multiple introductions of the virus or genetic evolution of a variant that has acted as an ancestor in autochthonous *H. lusitanicum* ticks in the Iberian Peninsula. Thus, adaptation of CCHFV to region-specific vectors and hosts leads to emergence of local virus variants as described recently in Kosovo (*12*). Genetic variability of these variants should be considered in molecular diagnostic methods (*39*).

The data obtained in this study, together with the CCHFV survey of ticks collected from animals conducted recently in Spain (*28*), as well as identification of 2 clinical cases caused by tick bites during 2016 and 2018 (*5*), show how the epidemiologic scenario of CCHF should include western Europe as an enzoonotic area and that surveillance studies on CCHFV in Spain and Portugal are necessary. Determining seroprevalence in animals and humans, as well as virus detection in ticks, would better define the risk for infection with CCHF in the Iberian Peninsula. These studies will help to determine the epidemiologic processes behind the known distribution of CCHFV in western Europe and establish adequate prevention and control measures to minimize the risk for cases of disease in Spain.
